# Evaluating the antioxidant potential of resveratrol-gold nanoparticles in preventing oxidative stress in endothelium on a chip

**DOI:** 10.1038/s41598-023-47291-6

**Published:** 2023-12-01

**Authors:** Farzaneh Fayazbakhsh, Fatemeh Hataminia, Houra Mobaleghol Eslam, Mohammad Ajoudanian, Sharmin Kharrazi, Kazem Sharifi, Hossein Ghanbari

**Affiliations:** 1 Department of Medical Nanotechnology, School of Advanced Technologies in Medicine, Tehran University of Medical Sciences, Tehran, Iran; 2 Department of Biotechnology and Molecular Medicine, Shahid Beheshti University of Medical Sciences, Tehran, Iran; 3 Research Center for Advanced Technologies in Cardiovascular Medicine, Cardiovascular Diseases Research Institute, Tehran University of Medical Sciences, Tehran, Iran

**Keywords:** Vascular diseases, Drug screening, Lab-on-a-chip, Nanobiotechnology, Cell biology, Microfluidics, Drug delivery

## Abstract

Vascular endothelial cells play a vital role in the health and maintenance of vascular homeostasis, but hyperglycemia disrupts their function by increasing cellular oxidative stress. Resveratrol, a plant polyphenol, possesses antioxidant properties that can mitigate oxidative stress. Addressing the challenges of its limited solubility and stability, gold nanoparticles (GNps) were utilized as carriers. A microfluidic chip (MFC) with dynamic flow conditions was designed to simulate body vessels and to investigate the antioxidant properties of resveratrol gold nanoparticles (RGNps), citrate gold nanoparticles (CGNps), and free Resveratrol on human umbilical vein endothelial cells (HUVEC). The 2, 2-diphenyl-1-picrylhydrazyl (DPPH) assay was employed to measure the extracellular antioxidant potential, and cell viability was determined using the Alamar Blue test. For assessing intracellular oxidative stress, the 2′,7′-dichlorodihydrofluorescein diacetate (DCFH-DA) assay was conducted, and results from both the cell culture plate and MFC were compared. Free Resveratrol demonstrated peak DPPH scavenging activity but had a cell viability of about 24–35%. RGNPs, both 3.0 ± 0.5 nm and 20.2 ± 4.7 nm, consistently showed high cell viability (more than about 90%) across tested concentrations. Notably, RGNPs (20 nm) exhibited antioxidative properties through DPPH scavenging activity (%) in the range of approximately 38–86% which was greater than that of CGNps at about 21–32%. In the MFC,the DCFH-DA analysis indicated that RGNPs (20 nm) reduced cellular oxidative stress by 57–82%, surpassing both CGNps and free Resveratrol. Morphologically, cells in the MFC presented superior structure compared to those in traditional cell culture plates, and the induction of hyperglycemia successfully led to the formation of multinucleated variant endothelial cells (MVECs). The MFC provides a distinct advantage in observing cell morphology and inducing endothelial cell dysfunction. RGNps have demonstrated significant potential in alleviating oxidative stress and preventing endothelial cell disorders.

## Introduction

Endothelial cells are important components of blood vessels, playing an important role in cardiovascular homeostasis by regulating blood flow and fibrinolysis, angiogenesis, monocyte adhesion, and platelet aggregation. The natural vascular endothelium is considered a protector of cardiovascular health^1^, while its abnormality is a major cause of many disorders including peripheral vascular disease, heart disease, diabetes, insulin resistance and chronic heart failure. Since an endothelial function can be a marker for the progression of these diseases, a more serious approach is needed to treat abnormal endothelial function^2^.

Studies have proven that oxidative stress mediates the production and secretion of cytokines in inflammation and endothelial dysfunction. Reactive oxygen species (ROS) are intermediate molecules that act as secondary messengers in the cell^3^. Increased ROS production causes aberrant regulation of many genes, including inflammatory cytokines and adhesion molecules. In response to a hyperglycemic environment, several proinflammatory pathways also participate in oxidative and antioxidant processes. As a result, an imbalance between enzymatic and non-enzymatic antioxidants and ROS generation leads to endothelial dysfunction, endothelial cell necrosis and apoptosis due to increased endothelial permeability^4^. ROS formation is associated with oxygen consumption and the action of oxidases during mitochondrial respiration. However, the accumulation of ROS until oxidative stress occurs is actually the result of a variety of factors. Most of these are inherent with physiological and biochemical processes that involve defects in glucose metabolism, usually due to hyperglycemia^5^. Hyperglycemia can destroy antioxidant protective enzymes, leading to an accumulation of ROS and subsequent cell damage^6^.

Chronic hyperglycemia has progressive detrimental effects on HUVECs, manifested by increased oxidative stress. The formation of free radicals is intensified by a decrease in antioxidant levels^7^ and results in oxidative damage, which ultimately leads to the dysfunction of endothelial cells^8,9^. Therefore, reducing oxidative stress is a crucial aspect of managing vascular diseases.

One of the effective strategies for the consequences of oxidative stress and diabetes-related hyperglycemia is the use of antioxidants, especially natural antioxidants^10^. Antioxidants have been extensively studied by neutralizing reactive oxygen/nitrogen species. There are many different natural ingredients that have antioxidant properties. In this study, we focus on resveratrol, a polyphenolic compound found in some plants such as grapes (specifically, red grape skins) and peanuts. Dietary resveratrol appears to act as an antioxidant and cardio protector. Recent studies have shown that resveratrol is a less toxic substance with a wide range of biological functions^11^. Resveratrol is a stilbenoid polyphenol with two phenolic rings connected by an ethylene bridge. The chemical structure of resveratrol (trans-3,5,4′-trihydroxystilbene) has been identified in two isomers: cis-resveratrol and trans-resveratrol. In Resveratrol the hydroxyl groups in the 3, 4′, and 5 positions are main factors in the antioxidant activity against hydroxyl (^·^OH) and hydroperoxyl (^·^OOH) radicals in aqueous^12^.

The hydrophobicity of polyphenols reduces their bioavailability, thereby limiting their therapeutic use^7^. With the help of nanotechnology and the use of nanoparticles as carriers, we can reduce these natural limitations, increase the bioavailability of polyphenols, and increase their effectiveness. Many nanoparticles and nanomaterials are manufactured as drug carriers^13^. GNps are one of the widely used metal carriers due to their biocompatibility and low relative toxicity. Studies have shown that GNps are candidates for antioxidant activity, preventing ROS formation, inhibiting free radicals, and increasing antioxidant-active defense enzymes in the body^8,9^. The use of GNps in treatment of skin inflammation^14,15^, cancer therapy^16^, tumoral vascular leakiness^17^ shows the wide application of these nanoparticles in medicine. Therefore, in this study, GNps were used as carriers to transport polyphenols and studied their antioxidant effects on endothelial cells.

A crucial aspect of treating cardiovascular diseases involves understanding the origins and mechanisms of these diseases within the dynamic environment of the body. In this regard, researchers have recently developed a more innovative method known as a MFC to recreate a better and more in vivo-like microenvironment for cells and tissues^18,19^. These devices can accurately simulate various parts of the body based on their specific designs. They can demonstrate the effects of different substances, drugs, and mechanisms^20^. These simulated platforms, by better simulating physiological conditions and better drug efficiency prediction using sophisticated in vitro models, have bridged the gap between data from animal and human studies^21–23^. MFC devices provide distinct advantages over conventional in vitro cell culture methods, by better regulating the micro environmental factors, such as fluid flow, shear stress, and gradients of nutrients and oxygen, that significantly impact the cellular behavior and the results of drug screening experiments^24,25^. Moreover, microfluidic cell culture systems offer several benefits for drug research, including fine control of the cellular microenvironment, and reduced reagent and cell consumption^26–28^.

**Figure 1:**
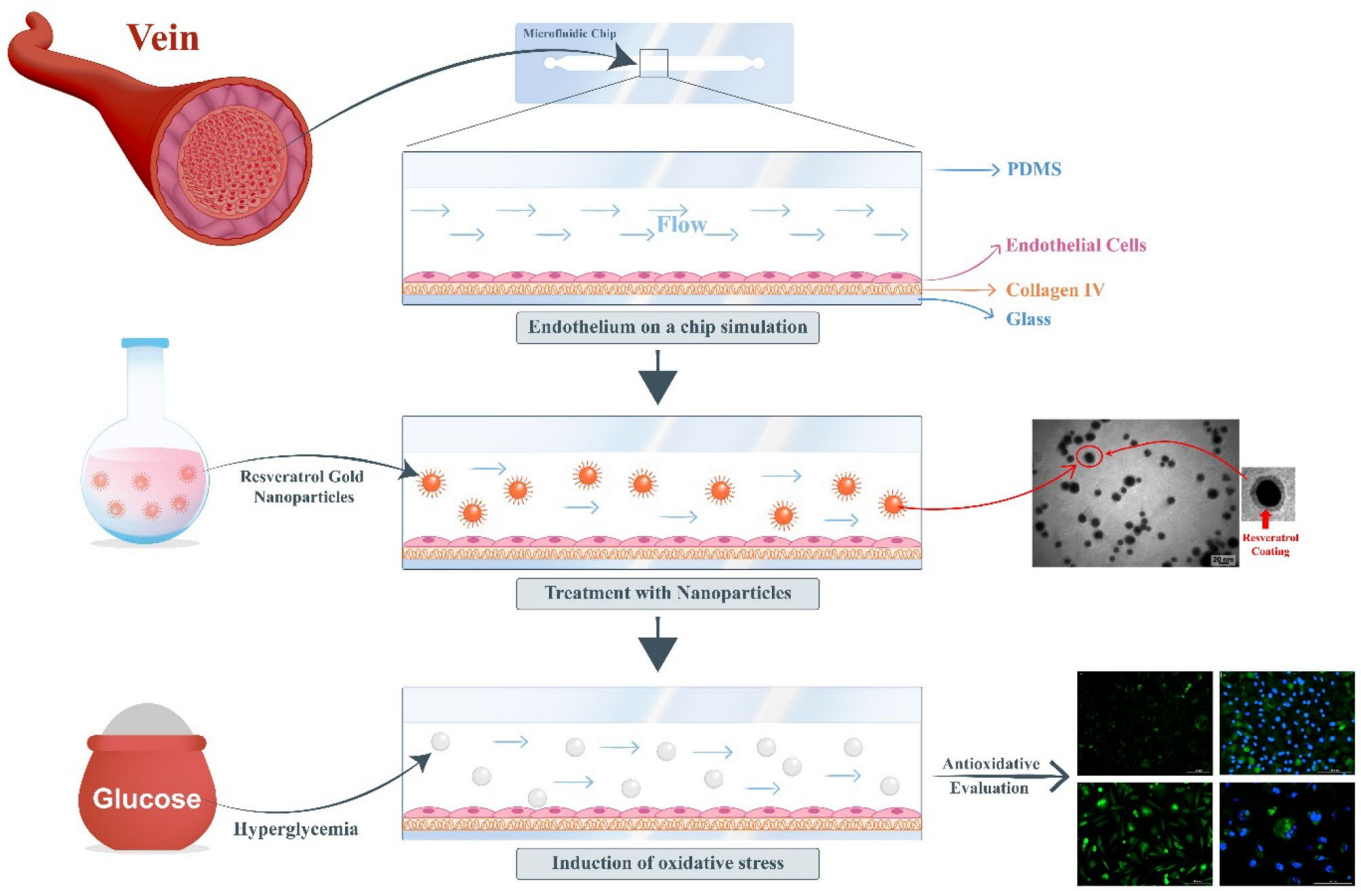
The primary stages of this research include the microfluidic fabrication process, the induction of hyperglycemia using glucose to trigger oxidative stress, and the evaluation of the antioxidant properties of Resveratrol gold nanoparticles (RGNps)' within a microchannel that mimics a vein.

The primary objective of this research, which represents its innovative aspect, is to investigate the antioxidant properties of RGNps in two different sizes (3 and 20 nm) within microfluidic chips (MFC) and to compare these properties with those in conventional cell cultures. After fabricating the chip and coating it with collagen type I, endothelial cell attachment on the microchannel surface was observed. This device as a vein simulator was used for a novel investigation method of GNps antioxidant properties. Oxidative stress was induced with high glucose concentration (hyperglycemia) in endothelial cells. The GNps were used as a carrier with antioxidant properties to transport resveratrol as an antioxidant. Finally, its effects on the cells cultured both on the MFC and conventional cell culture plates have been studied and compared. The main steps of this study were performed in Fig. 1.

## Materials and methods

### Synthesis of GNps

RGNps with sizes of approximately 3.0 ± 0.5 nm and 20.2 ± 4.7 nm were synthesized using the following procedure. Various sizes of NPs were prepared by synthesizing them at different concentrations of Resveratrol (Sigma-Aldrich) and NaOH (Sigma-Aldrich). As pH is one of the factors that control the synthesis of GNPs, it was adjusted using NaOH solution. Also, the Turkewich method was used to make CGNps at approximately 20 nm. The NPs were then washed four times with an Amicon filter (50 kDa, Merck Millipore) through centrifugation at 4000 rpm for 5 min.

#### Synthesis of RGNps (3 nm and 20 nm)

Initially, 4 ml of an aqueous gold solution (1.25 mM, HAuCl_4_.3H_2_O, 99% from Sigma-Aldrich) was added to a round-bottom flask and placed on a magnetic stirrer operating at 1500 rpm. Subsequently, 1 mg of resveratrol was dissolved in 1 ml of a 0.02 M NaOH solution in the dark. This solution was immediately transferred to the flask, followed by the immediate addition of 2 ml of 0.02 M NaOH for the 3 nm RGNps until the color change from yellow to red was complete. For the 20 nm RGNps synthesis, 700 μL of NaOH (0.02 M) was added to the reaction mixture instead, resulting in a color change from yellow to purple, confirming the formation of gold nanoparticles.

#### Synthesis of CGNps

First, 24.3 ml of the gold solution (0.27 mM) was added to a flask and stirred at 1000 rpm while increasing the temperature until the water was completely boiling. Then, 700 µL of sodium citrate solution (15%, Sigma-Aldrich) was added to the flask, changing the color from yellow to red.

### Design, fabrication, and surface modification of MFCs

The design of the microfluidic system depended on the cell line used for the assay. After selecting the design based on HUVEC and vein simulation, the desired mold was first fabricated using a photolithographic method. The MFCs were then fabricated using soft lithography and the surface was modified according to facilitate HUVEC cell attachment. The steps are described in full as follows:

#### Photolithography

**Figure 2:**
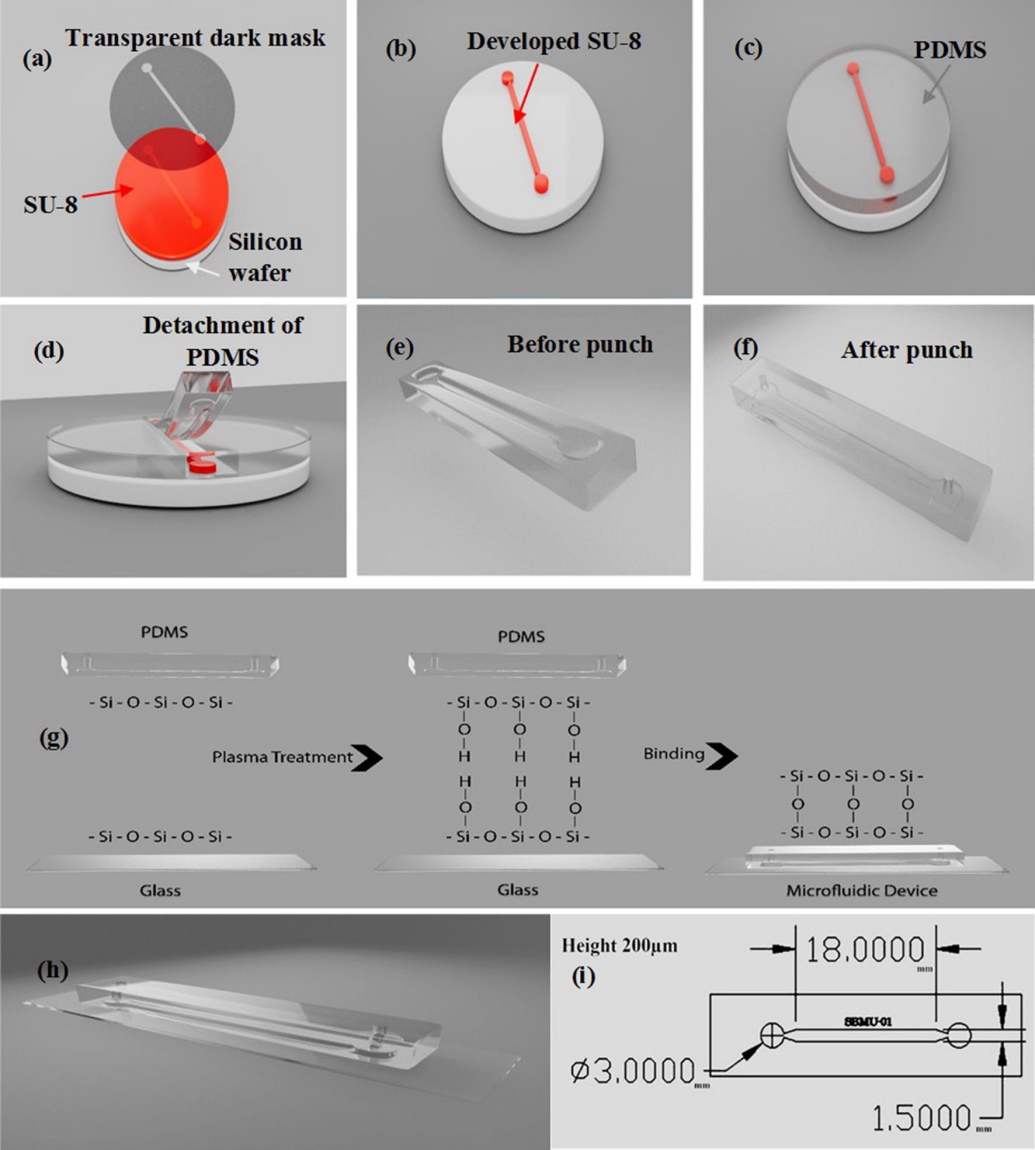
Chip fabrication steps, the schematic image of photolithography (**a**–**c**), the schematic image of soft lithography steps (**d**–**f**), the schematic image of PDMS (Polydimethylsiloxane) surface and glass during plasma bonding (**g**), the final schematic (**h**), and channel dimensions (**i**).

The desired pattern and dimensions were designed using AutoCAD software to prepare the mask file for lithography. A silicon wafer was used as the substrate and coated with SU-8 (Fig. 2a), a photoresist material. The mask was then placed on the silicon wafer coated with SU-8 to achieve the desired thickness (Fig. 2b). This was done at a proportional speed, following the standard diagrams for SU-8 spin coating. The silicon wafer was subsequently heated on a hot plate at a temperature of 65 °C, and then placed on a hot plate at 95 °C for 15 min. It was exposed to UV light for 400 s, depending on the operating power of the lamp. After the previous heating, it was placed on the hot plate again at 95 °C for 12 to 15 min to perform the post-baking step. Finally, the mold was placed in a developer-curing solution to wash away the unexposed portions. At this stage, the desired pattern was obtained^29–33^.

#### Soft lithography

For casting, PDMS (Polydimethylsiloxane, Dow Corning 184 Sylgard Silicone) in a ratio of 10 to 1 w/w was thoroughly mixed with its cross-linker solution and placed in a desiccator. The vacuum pump was connected to the desiccator and suctioned for 20 min to remove bubbles in the PDMS. PDMS was poured into the mold and placed in the oven at 75 °C for 3 h (Fig. 2c). Finally, the solid PDMS was removed from the mold (Fig. 2d,e) and punched to make the input and output of the channels (Fig. 2f) using a biopsy punch of the dimensions of 1.5 mm^29^.

#### Bonding procedure

The Oxygen plasma bonding method was used to activate and attach PDMS to the glass which was the bottom surface of the chip. PDMS surface was oxidized and activated by plasma oxygen (O_2_, Harrick Plasma cleaner, USA,). Oxygen plasma removes organic matter and hydrocarbons by chemical reaction with highly reactive oxygen radicals and erosion by energetic oxygen ions. This allows silane (Si–OH) groups to remain on the surface, making the surface more hydrophilic and increasing the surface wettability. When the plasma is activated, the PDMS is immediately in contact with the oxidized PDMS or another glass surface to form a Si–O-Si bond on the joint surface to form an irreversible seal. This water-resistant covalent bond is appropriate for microchannel formation. In order to do this, the PDMS and the glass were placed in the plasma cleaner for 70 s in which their surface was exposed to direct radiation. It was immediately removed from the device and placed on top of each other to complete the connection (Fig. 2g). The chips were then placed in the oven at 70 °C for 2 h for better adhesion^29^. At the end of this step, the desired chips were completely made (Fig. 2h,i).

#### Surface modification

Collagen (I) (Extracted from the tail mouse) has been used to improve the adhesion of cells to the surface after repair by plasma binding, especially under current exposure conditions. After chip sterilization with 70% ethanol and UV irradiation for 20 min, collagen was dissolved in phosphate-buffered saline (PBS, Gibco) at a concentration of 0.05 M^34^, injected into the channels, and re-irradiated with UV light for 20 min to cross-link the collagen. The chip was then placed in an incubator for 24 h to form a bio-adhesive layer at the bottom of the channel.

### Measurement of the extracellular antioxidant effect of nanoparticles

DPPH (Sigma-Aldrich) was used to measure the antioxidant efficacy of nanoparticles prepared in an extracellular medium. DPPH is a crystalline compound that acts as a stabilizing free radical as well as a radical receptor. DPPH color changed from dark purple to yellow or colorless after neutralization In DPPH inhibition calculation, for UV absorbance overlap of gold nanoparticles, was subtracted from the absorbance of GNps with DPPH at 517 nm.

### Cell culture procedure

In this study, human umbilical vein cells (HUVEC, Pasteur Institute's genetic resource center) were used. The cell culture medium for these cells was High Glucose DMEM (4.5 g/L Glucose, Gibco-BRL). The treated cells were divided into groups receiving RGNps at 3 nm and 20 nm, CGNps at 20 nm, and free Resveratrol. The study groups included a positive control post-treated with extra glucose (100 mM, Sigma-Aldrich Aldrich) to induce hyperglycemia after GNps treatment. One group without any treatment was considered as a negative control. The primary evaluations in this study were cytotoxicity and antioxidant effects assays of the GNps in conventional cell culture.

#### Cell viability assay in the cell culture plate

HUVEC cells were seeded on a 96-well cell culture plate with a certain volume of medium containing approximately 5,000 cells/well and incubated in humidified air with 5% CO_2_ at 37 °C. Cells were treated triplicate in 4 groups: RGNps at about 3 and 20 nm, CGNps, and free Resveratrol at 6 different concentrations (25, 50, 100, 150, 200, 250 ppm) with positive and negative control groups.

After 24, 48 and 72 h, Alamarblue solution (10%) with culture medium was added to each well. Then using an ELISA reader (Cytation 3), the absorbance of each well was measured at 570, and 600 nm and, cell viability was calculated.

#### Cell culture in MFC

The cell suspension was prepared at a density of 2 × 10^6^ cells/mL suitable for injection into the channel of MFC. Accordingly, the desired amount of suspension containing cells was injected into the channel. MFCs were then placed in sterile petri dishes and incubated in a cell culture incubator with 5% CO_2_ at 37 °C for 24 h. By using a sterile syringe and a sterile tube containing a complete cell culture medium, the chip inlet and outlet were successfully connected. A flow rate of 1 µL/min was applied to induce dynamic conditions inside the chip. After 48 h, the channels had reached an appropriate confluence of about 70–80%. At this stage, the chips were treated with different concentrations of testing groups containing fresh culture medium for 24 h.

#### Cell treatment with glucose and oxidative stress-inducing

After 24 h of GNps treatment, hyperglycemia was induced in both plates and chips for all groups except the negative control by treating with a culture medium containing 100 mM glucose for 24 h.

#### Evaluation of the antioxidant effect of nanoparticles on the cells in MFC and plate using DCFH-DA assay

In this study, the fluorescence detector DCFH-DA (2%, Sigma-Aldrich) was used to investigate the antioxidant effect of nanoparticles on cellular oxidative stress. The DCFH-DA is a fluorescent probe used to detect the presence of various ROSs such as hydrogen peroxide, hydroxyl radicals, and peroxynitrite. This substance enters the cells through an active diffusion mechanism and is converted to DCFH by cellular esterase. In the presence of intracellular ROS, DCFH is then converted to DCF, which has fluorescence properties (with an excitation wavelength of 485 nm and emission wavelength of 530 nm) and can be observed with fluorescence detection instruments. The amount of fluorescence emitted from the cells by ROS produced can be measured with a flow cytometer, ELISA reader, and other fluorescence microscopes^35^.

The effect of nanoparticles at different concentrations on the amount of oxidative stress was investigated using the Cytation instrument (Olympus) and its fluorescence microscopy. After cell incubation and treatment with glucose the culture medium was removed from the cells and the cells were washed with PBS. DCFH-DA solution, previously prepared by dissolving in DMSO (Sigma-Aldrich), was prepared in 10 μm of FBS-free cell culture medium. In each well of a 96-well cell culture plate, 100 μL was added, and for MFC evaluation, approximately 30–40 μL was injected into the channels of the chip. The cells were incubated in the dark for 30 min. Then, the medium was removed from the cells and the cells were washed twice with PBS. Finally, 100 µL per well was added to the 96-well cell culture plates, and approximately 30–40 µL of PBS was injected into the chip channels. For staining of the cell nuclei, a solution of 4′,6-diamidino-2-phenylindole (DAPI) prepared in PBS was injected into each channel. In this step, the cells were examined using fluorescence microscopy, and the fluorescent intensity was measured with ImageJ.

#### Statistical analysis

The data obtained was analyzed by GraphPad prism 6 software using One-Way ANOVA, and the corresponding p-values are indicated in the diagrams using stars, with significance levels shown as follows: **p < 0.01, ***p < 0.001, and ****p < 0.0001.

## Results and discussion

### Synthesis and characterization of RGNps

During the process of synthesizing GNps with Resveratrol, a distinct color change in the solution was evident. This transformation, often associated with the surface plasmon resonance (SPR) of nanoparticles, signifies the successful synthesis of GNps^36^. Resveratrol served multiple roles in this synthesis: as a reducing agent, a stabilizer, and an antioxidant. This multi-functionality not only confirms the successful synthesis of GNps but also indicates the effective loading of resveratrol onto these nanoparticles.

**Figure 3:**
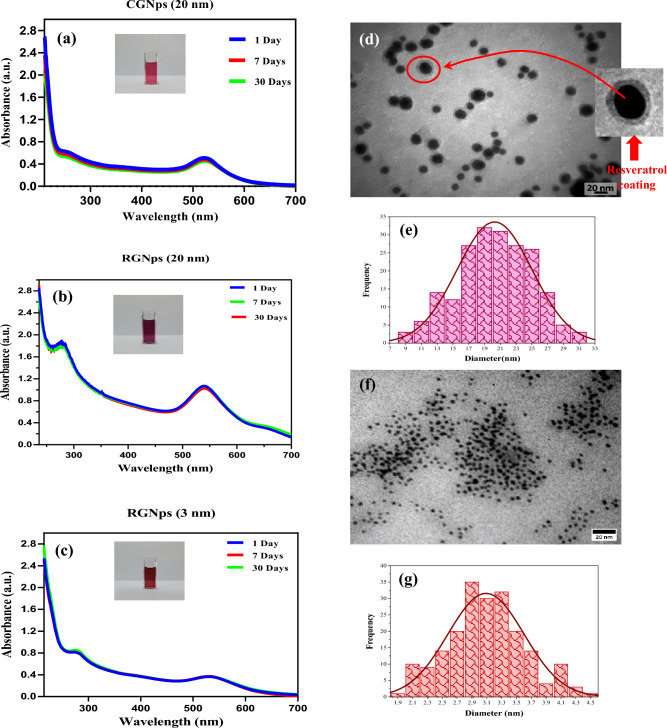
The UV absorbance spectrum of citrate gold nanoparticles (CGNps) at 20 nm (**a**) the Ultra-violate (UV) absorbance spectrum of RGNps (20 nm) (**b**), the UV absorbance spectrum of RGNps (3 nm) (**c**), transmission electron microscopy (TEM) Images of Resveratrol gold nanoparticles (RGNps) with size distribution histogram (20.2 ± 4.7 nm) (**d**,**e**), TEM Images of RGNps with size distribution histogram (3.0 ± 0.5 nm) (**f**,**g**).

Subsequent spectrophotometric examinations of both RGNps in two dimensions and CGNps confirmed the presence of the UV absorbance peak characteristic of GNps. Specifically, Fig. 3a illustrates the UV absorbance peak of CGNps (20 nm) after 30 days, as captured by a UV spectrometer (Perkin Elmer, USA). Similarly, Fig. 3b,c display the UV absorbance of RGNPs for sizes 20 and 3 nm, respectively, after the same duration. These observations underscore the stability of the synthesized nanoparticles. Furthermore, the estimated size of CGNps was deduced to be approximately 20 nm, based on the wavelength of its UV absorbance^37^.

### Transmission electron microscopy results

Transmission electron microscopy (TEM) images (Zeiss EM10C, Germany) revealed that RGNps exhibited sizes of 20.2 ± 4.7 nm (Fig. 3d) and 3 ± 0.51 nm (Fig. 3 f). These particles displayed a spherical and uniform distribution, as evident from the histograms. Such uniformity in nanoparticle size and shape is crucial, especially when considering their potential therapeutic applications and biodistribution. By analyzing 200 nanoparticles, the average size was determined, with the normal distribution presented in Fig. 3e,g.

### DLS analysis and zeta potential results

Dynamic light scattering (DLS) provides insights into the hydrodynamic diameter of nanoparticles. This diameter can differ from their actual size due to the hydration shell. The zeta potential, on the other hand, is indicative of the stability of the nanoparticle suspension. Using DLS, the hydrodynamic diameter of RGNps (20 nm) was determined to be about 62.4 nm, while for RGNps (3 nm), it was measured at 25.5 nm. The hydrodynamic diameter of the CGNps (20 nm) was found to be approximately 34.4 nm (Supplementary File, Fig. 1). Furthermore, the zeta potential analysis of RGNps at 3 and 20 nm showed − 46.4 ± 3 and − 50.7 ± 0.9 mv, respectively (Supplementary File, Fig. 2). DLS measurements can be larger than TEM measurements due to the accumulation of water molecules on the charged and coated surface of the nanoparticles, which increases the hydrodynamic diameter. The zeta potentials obtained from RGNps indicate the high colloidal stability of these particles in an aqueous solution.

### Implications of GNps size

The biodistribution and clearance of nanoparticles are influenced by various factors including their size, shape, material composition, biodegradability, and surface modifications. Previous studies have shown that nanoparticles smaller than 5–10 nm are prone to rapid renal clearance because they can easily pass through the kidney's filtration system^38^. On the other hand, to reduce uptake by the mononuclear phagocytic system, nanoparticles should ideally be smaller than 100 nm^39^.

The study predominantly focuses on the 20 nm nanoparticles, chosen for their optimal therapeutic range. Additionally, the 3 nm size was investigated to assess if a smaller size, with a greater surface-to-volume ratio, might offer enhanced potency. If the hydrodynamic size be considered both the 3 nm and 20 nm nanoparticles demonstrated acceptable hydrodynamic sizes as determined by DLS, indicating their suitability for certain biomedical applications. However based on actual size taken by TEM, the 3 nm GNps, might have cleared by kidney due to their small size, while the 20 nm GNps might offer a balance between cellular uptake and prolonged circulation.

While there are guidelines for nanoparticle size, their behavior in the bloodstream varies widely in studies. This inconsistency is due to the vague definition of "clearance efficiency" and the many factors, like material, size, shape, and charge that affect how the body processes them^38^.

### Results of cell viability in the cell culture plate

**Figure 4:**
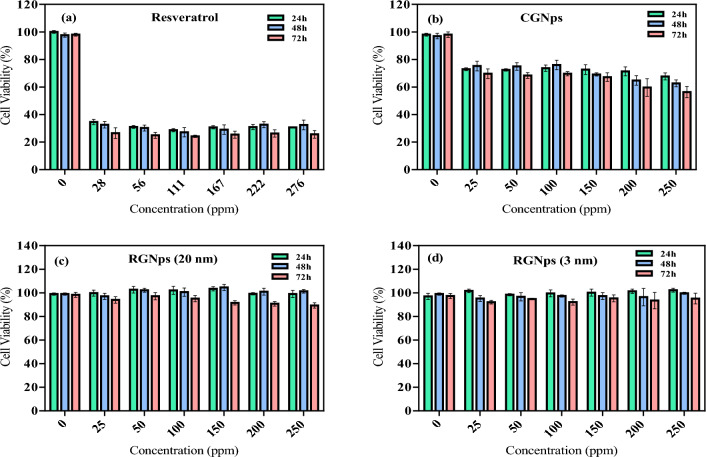
Investigating the survival of endothelial cells after treatment on cell culture plate at 24, 48 and 72 h with free Resveratrol (**a**), citrate gold nanoparticles (CGNps) (**b**), Resveratrol gold nanoparticles (RGNps) (20 nm) (**c**) and RGNps (3 nm) (**d**), control groups represented in concentration of 0 ppm, the number of experiment (n = 3).

Cell viability assays, such as the Alamar Blue assay, are pivotal for quantitatively assessing cell health and proliferation. This is especially vital when evaluating the biocompatibility of GNps. In the study, the cell viability at various concentrations (25, 50, 100, 150, 200, and 250 ppm) of free Resveratrol, CGNps, RGNps (20 nm), and RGNps (3 nm) was examined using the Alamar Blue assay at intervals of 24, 48, and 72 h (Fig. 4). It was observed that free Resveratrol, across all tested concentrations over the span of 1, 2, and 3 days, exhibited low cell viability ranging between 24 and 35% (Fig. 4a) and presented undesirable cell morphology in the culture (Fig. 6e). Notably, at higher concentrations, free Resveratrol dissolved in the culture medium during treatment but recrystallized overnight, indicating significant cytotoxicity. In contrast, RGNps of both 3 nm and 20 nm sizes demonstrated high cell viability across all concentrations and time frames in the cell culture. This suggests that using GNps as a carrier effectively reduced the toxicity of Resveratrol at all concentrations, potentially due to enhanced hydrophilicity and antioxidant activity.

### DPPH assay results

The extracellular antioxidant properties of RGNps were evaluated using the DPPH assay, which measures the degree of radical inhibition. Absorbance at 517 nm was used to determine the scavenging activity of each sample across six concentrations, as described by Eq. (1). For better analysis, the antioxidant properties of RGNps were evaluated in comparison to CGNps at the same concentrations and free Resveratrol based on the calculated Resveratrol content of the RGNps (20 nm). DPPH scavenging activity (%) was calculated from the following equation.

1 $$DPPH \; scavanging \; activity \; (\mathrm{\%})=\frac{{OD}_{0}-({\mathrm{OD}}_{1}-{OD}_{GNps})}{{\mathrm{OD}}_{0}}\times 100$$

where OD_0_ is the DPPH absorbance of the control sample, OD_1_ is the DPPH absorbance of the test samples and OD_GNps_ is the UV absorbance of GNps at the same concentration in the test sample without DPPH at a wavelength of 517 nm.

**Figure 5:**
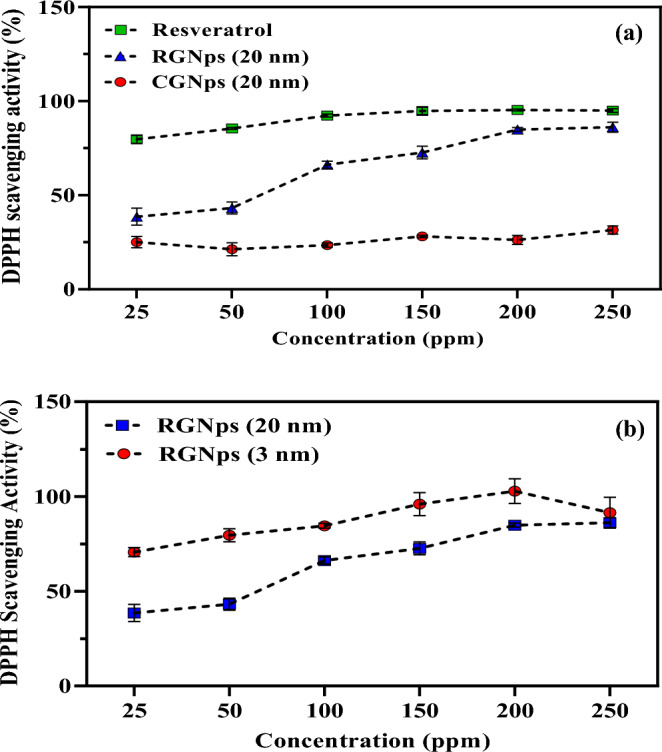
Inhibition of 2, 2-diphenyl-1-picrylhydrazyl (DPPH) (%) for extracellular antioxidant activity, for Resveratrol gold nanoparticles (RGNps) (20 nm) in comparison with citrate gold nanoparticles (CGNps) (20 nm) and free Resveratrol (**a**), for RGNps (20 nm) in comparison with RGNps (3 nm) (**b**), n = 3.

According to the following graph in Fig. 5a, the antioxidant properties of RGNps were increased by increasing concentrations. Compared to CGNps, RGNps have shown a significant antioxidative effect. For example, at 150 ppm, RGNps showed about 2.5 times the antioxidative property. In contrast to free Resveratrol, at lower concentrations, they have less scavenging properties. Based on some studies, while polyphenol-gold nanoparticle complexes have been shown to have some benefits in terms of stability and bioavailability, their antioxidant activity may be lower compared to free polyphenols at the same concentration. Possible explanations for this phenomenon include the stabilization effect, changes in molecular structure, nanoparticle size, and method of preparation^40,41^. However, as the concentrations were increased, the oxidative effect also increased. Also, at high concentrations, RGNps showed similar antioxidant properties to free Resveratrol.

In addition, as shown in Fig. 5b, RGNps (3 nm) have shown more antioxidant activity than RGNps (20 nm), which might be caused by a higher loading of Resveratrol and a high surface-to-volume ratio. In fact, as the size decreased to 3 nm, the volume-to-surface ratio increased, and more Resveratrol was loaded onto the nanoparticles. As a result, Resveratrol increased antioxidant effect. However, there were no differences between the oxidative scavenging properties of both sizes of RGNps and Resveratrol at 250 ppm. This indicates the antioxidant property reached a saturated level, and further increasing in concentration had no significant changes in antioxidant properties.

### The observation of different morphology in the MFC *vs* cell culture plate

**Figure 6:**
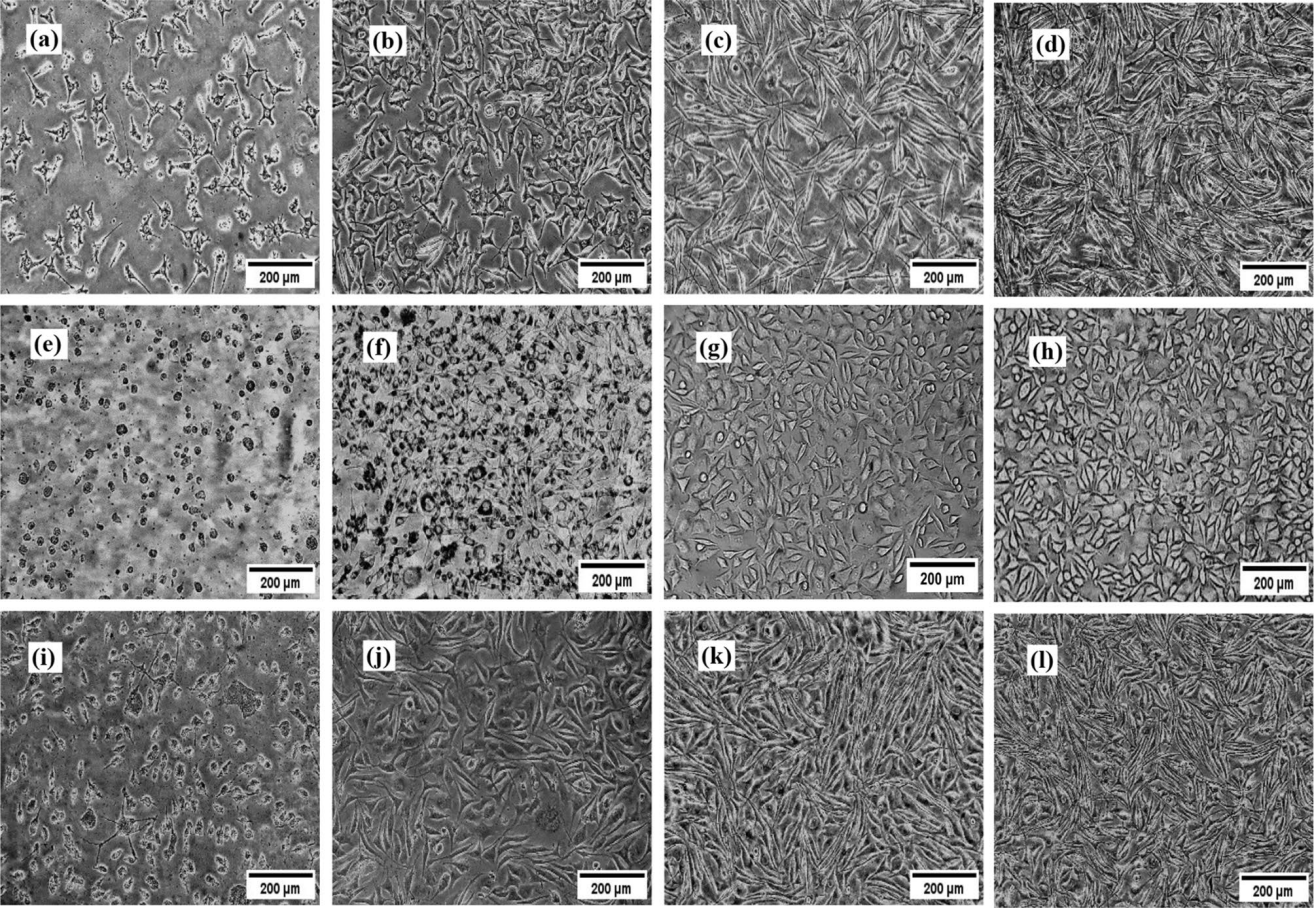
The cell morphology after 1 and 3 days on the cell culture plate (**a**,**b**), and microfluidic chip (MFC) (**c**,**d**), light microscopic image from human umbilical vein endothelial cells (HUVEC) cells in the cell culture plate after 24 h treatment with free Resveratrol (**e**), citrate gold nanoparticles (CGNps) (**f**), Resveratrol gold nanoparticles (RGNps) (20 nm) (**g**) and RGNps (3 nm) (**h**) at 100 ppm, light microscopic image from HUVEC cells in MFC after 24 h treatment with free Resveratrol (**i**), CGNps (**j**), RGNps (20 nm) (**k**), and RGNps (3 nm) (**l**), at 100 ppm.

After incubating the cells in a cell culture incubator, the MFCs were examined using a light microscope. By comparing the cells cultured in the MFC with those in the cell culture plate at intervals of 1 to 3 days after injection, HUVECs on the MFC (Fig. 6c,d) achieved better morphology than the cell culture plate (Fig. 6a,b) in less time. Indeed, the cells in the MFC have a more stretched morphology compared to the cell culture plate. Generally, endothelial cells are referred to as polygonal and cubical stone cells. However, this morphology can be changed in dynamic flow from a polygonal to a more stretching shape^42^. Under physiological shear stress conditions, vascular endothelial cells along the vessels were formed in the direction of parallel and spindle flow. In fact, these cells changed their morphology from cubical stone to spindle-like in response to the dynamic flow. This mechanotransduction played an important role in regulating morphology, function and a wide range of cell signals between the vascular system and the surrounding tissue^43^. Furthermore, the response of endothelial cells to directional flow led to the remodeling of cell structures, which minimized intracellular stress through the use of adaptive signals. These morphological changes were accompanied by cytoskeletal reorganization with the rearrangement of actin filaments. As a result, these morphological and functional changes in endothelial cells protect the vessels by maintaining vascular homeostasis. Based on research, endothelial cells aligned and elongated under dynamic flow conditions, which improved their function in maintaining blood vessel integrity, regulating blood flow, and responding to biochemical signals. This contributed to the proper function of the cardiovascular system^44,45^. Microscopic images of the endothelial cells in the plate and MFC revealed differences in cell morphology.

### Microscopic examination of cells after treatment in MFC* vs* cell culture plate

The HUVEC cells after treatment with free Resveratrol, CGNps and RGNps (20 nm and 3 nm), at a certain concentration are shown in Figs. 6e–h for the cell culture plate and in Figs. 6i–k and l for the MFC. The cells treated with Resveratrol showed cell death, loss of their morphology and the removal of a significant number of them in both platforms. Also, after cell treatment with CGNps, by mixing the nanoparticles with the cell culture medium, due to the instability of the nanoparticles, gold deposition was seen on the cells. This phenomenon was too intensive in the cell culture plate. However, the accumulation and deposition of CGNps (Fig. 6f *vs* j) and recrystallization of Resveratrol (Fig. 6e *vs* i) on the cells in MFC were much less than on the cell culture plate. In MFC, unlike common cell culture plates, there were dynamic conditions and fluid flow. As a result of dynamic flow, the risk of excessive accumulation of substances in the cells was lower than the static circumstances.

### Inducing MVECs by hyperglycemia in MFC

The observation of MVECs among the cells after inducing hyperglycemia in the cell culture showed that endothelial dysfunction was successfully induced.

However, after inducing hyperglycemia in MFC and cell culture plate staining with DCFH-DA and DAPI, some giant endothelial cells with abnormal morphology and multinucleated structure were observed just in the MFC system. Studies have shown that high glucose levels can play a role in the development of MVECs. This appeared due to the impairment of endothelial cell function, activation of pathways that promoted cell fusion, increased production of reactive oxygen species, and the up-regulation of proteins that encouraged cell fusion. Therefore, endothelial cell dysfunction occurred leading to the formation of MVECs^46,47^. These cells which were associated with atherosclerosis were larger in size and multinucleated morphology with the loss of original function^48–51^.

**Figure 7:**
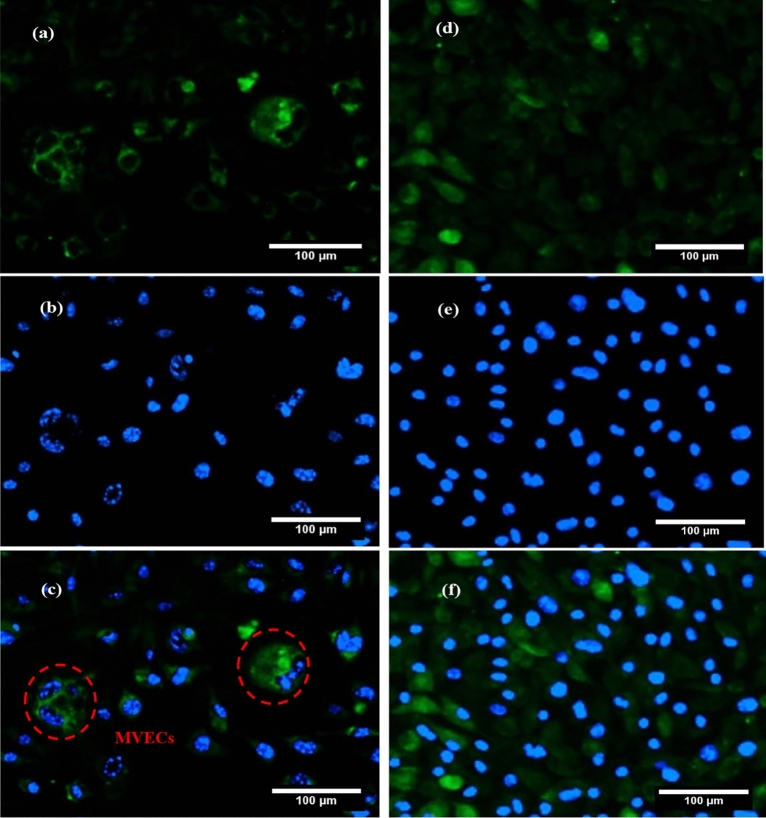
The fluorescent Images of some multinucleated variant endothelial cells (MVEC) cells in microfluidic chip (MFC) after the staining by 2′,7′-dichlorodihydrofluorescein diacetate (DCFH-DA) (**a**) and 4′,6-diamidino-2-phenylindole (DAPI) (**b**) and merged image (**c**) under hyperglycemic conditions. The fluorescent Images of the cells in cell culture plate after the staining by DCFH-DA (**d**) and DAPI (**e**) and merged image (**f**) under hyperglycemic conditions.

The fluorescent images of MVECs in MFC after the fusion of nuclear (DAPI) and cytoplasmic (DCFH-DA) staining have been shown under hyperglycemic conditions. The cell with the multinucleated structure was visible in Fig. 7a–c. The fluorescent images of the cell culture plate after the fusion of nuclear (DAPI) and cytoplasmic (DCFH-DA) staining have been shown under hyperglycemic conditions. The multinucleated structure was not found in Fig. 7d–f.

### Investigating the intracellular antioxidant effect of DCFH-DA

**Figure 8:**
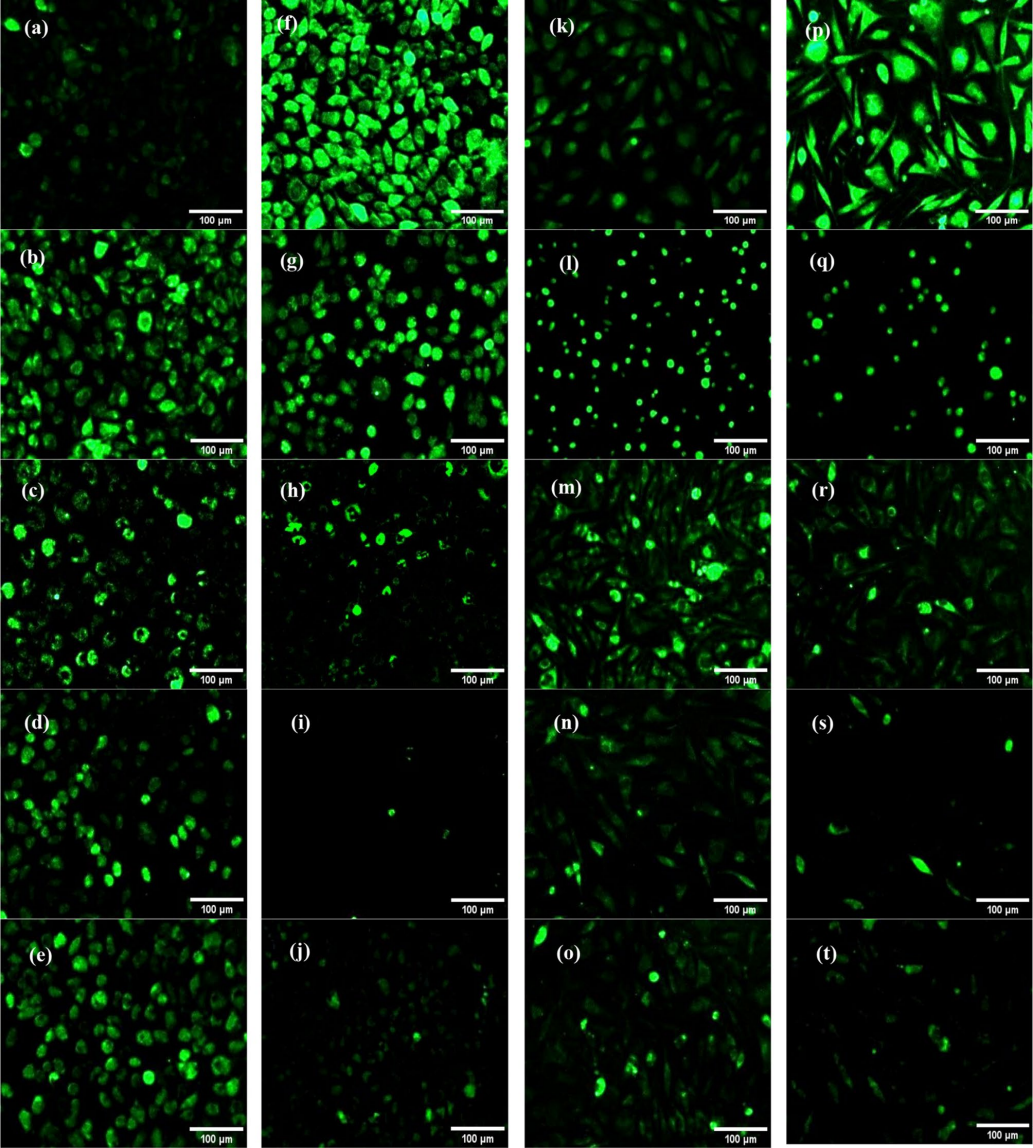
Fluorescence microscopic images of 2',7'-dichlorodihydrofluorescein diacetate (DCFH-DA) staining for human umbilical vein endothelial cells (HUVEC) in cell culture plate before (**a**) and after high glucose treating (**f**), free Resveratrol at 28 ppm (**b**) and citrate gold nanoparticles (CGNps) (**c**), Resveratrol gold nanoparticles (RGNps) (20 nm) (**d**) and RGNps (3 nm) (**e**) at 50 ppm and free Resveratrol at 222 ppm (**g)** and CGNps (**h**), RGNps (20 nm) (**i**) and RGNps (3 nm) (**j**) at 200 ppm, fluorescence microscopic images from HUVEC cells in MFC before (**k**) and after high glucose treating (**p**), free Resveratrol at 28 ppm (**l**) and CGNps (**m**), RGNps (20 nm) (**n**) and RGNps (3 nm) (**o**) at 50 ppm and free Resveratrol at 222 ppm (**q**) and CGNps (**r**), RGNps (20 nm) (**s**) and RGNps (3 nm) (**t**) at 200 ppm.

Figure 8 showcases the intracellular antioxidant properties of Resveratrol and all GNps groups at concentrations of 50 and 200 ppm, as indicated by the DCFH-DA fluorescent indicator. The fluorescent microscopic images (BioTek, USA) revealed that RGNps, in both sizes, significantly reduced cellular oxidative stress. This reduction can be attributed to the inherent antioxidant properties of Resveratrol, which, when combined with the GNps, may have facilitated a more efficient cellular uptake and distribution, leading to enhanced antioxidative effects.

**Figure 9:**
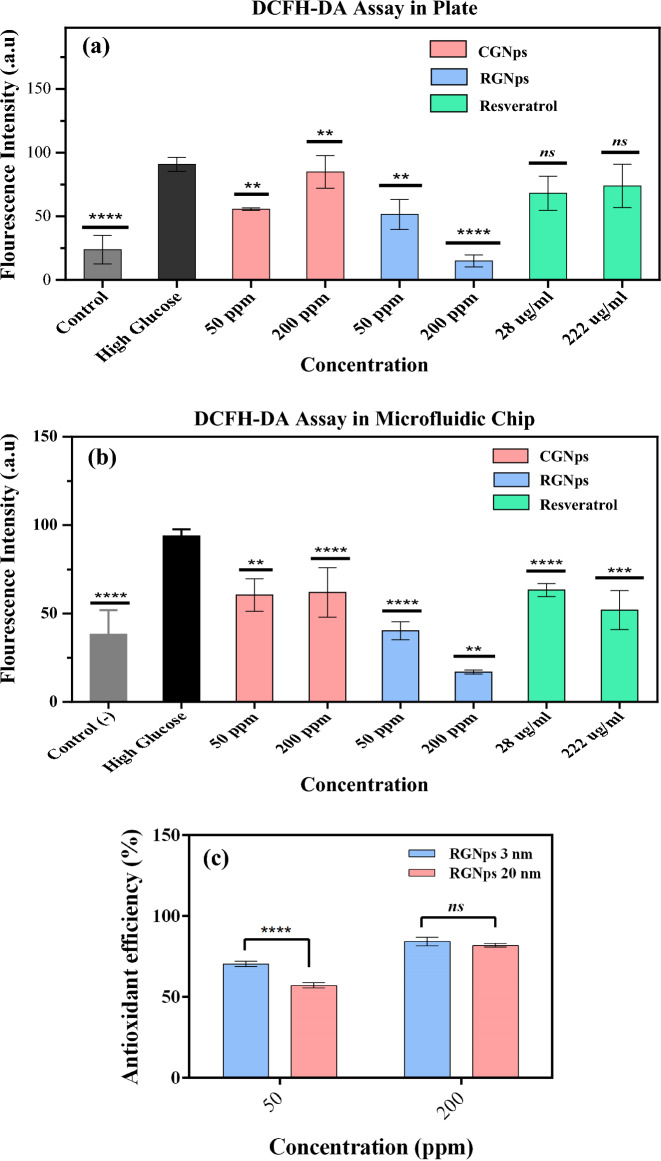
Investigation of the level of oxidative stress of citrate gold nanoparticles (CGNps), Resveratrol gold nanoparticles (RGNps) (20 nm) at 50 and 200 ppm and Resveratrol at 28 and 222 µg/mL on endothelial cells in the microfluidic chip (**a**) and in the cell culture plate (**b**) Investigation of the level of oxidative stress on endothelial cells for Resveratrol gold nanoparticles (RGNps) at 3 and 20 nm, with a stress reduction of 70.4 ± 1.6% and 57.1 ± 1.5% at 50 ppm and, with reductions of 84.3 ± 2.5% and 81.9 ± 1.2% at 200 ppm, respectively (**c**), n = 3 and significance levels shown as follows: **p < 0.01, ***p < 0.001, and ****p < 0.0001.

The quantitative analysis, as depicted in Fig. 9a,b, highlighted discernible differences between the MFC and the cell culture plate. In the MFC, CGNps reduced cellular oxidative stress by 34–35.5% under hyperglycemic conditions, while Resveratrol achieved a reduction of 32–44.7%. RGNps (20 nm), however, exhibited a more pronounced effect, reducing oxidative stress by 57–82%. In the cell culture plate, the reductions were 6.6–38.7% for CGNps, 18.7–25% for Resveratrol, and 43.3–83.5% for RGNps (20 nm).

The superior performance of RGNps can be attributed to their ability to enhance the bioavailability of Resveratrol, ensuring its effective delivery to cells. This is further evidenced by the observation that cells treated with RGNps in the MFC at 50 ppm exhibited a more pronounced reduction in oxidative stress compared to the same concentration in the cell culture plate. Similarly, the reduced accumulation of Resveratrol crystals and deceased cells in the MFC led to a more significant antioxidative effect for free Resveratrol and CGNps at 200 ppm. Supporting these results, gold-conjugated polyphenol nanoparticles synthesized through ecofriendly methods have been proposed as suitable carriers for bioactive polyphenols, offering therapeutic potential against various disorders associated with oxidative stress^52^. Also, recent studies have highlighted the antioxidative effects of Resveratrol. For instance, the free radical scavenging ability of Resveratrol has been extensively documented, with its antioxidative properties being attributed to various mechanisms, including signaling pathways and the regulation of antioxidant enzyme^53^. Another study emphasized the hepatoprotective and therapeutic effects of Resveratrol, particularly its anti-inflammatory and antioxidative activities^54^.

### Comparing the antioxidative effect of RGNps at 3 and 20 nm in MFC

The antioxidative effect of RGNps in 3 and 20 nm at 50 and 200 ppm was compared by DCFH-DA assay in the MFC (Fig. 9c). The result showed that RGNps (3 nm) and RGNps (20 nm) had similar efficacy in reducing oxidative stress, with reductions of 84.3 ± 2.5% and 81.9 ± 1.2% respectively, at 200 ppm. However, at 50 ppm, RGNps (3 nm) demonstrated greater efficacy, with a stress reduction of 70.4 ± 1.6%, compared to RGNps (20 nm), which showed 57.1 ± 1.5% efficacy. This might be due to the smaller size allowing for more Resveratrol loading. The surface of nanoparticles presented potentially active sites of functional groups for reacting to another reagent. Smaller-sized nanoparticles have a larger surface-to-volume ratio. Therefore, the smaller GNps could deliver and present more Resveratrol functional groups to oxidative stress species.

### Non-animal models in drug screening research

Recent studies have emphasized the importance of a multi-dimensional approach to understanding drug delivery mechanisms and their therapeutic implications. This approach integrates in vitro, ex vivo, and in vivo methods^55^. In vivo studies provide a comprehensive understanding of the pharmacokinetics, biodistribution, and potential side effects of therapeutic agents in a living system. On the other hand, non-animal models have been highlighted for their significance in drug permeation studies. These models offer insights into drug delivery mechanisms without the ethical concerns associated with animal testing^56^.

The primary aim of this study was to utilize a microfluidic chip (MFC) to simulate body vessels as a non-animal model and investigate the antioxidant properties of RGNps under controlled conditions. The MFC enabled close monitoring and evaluation of the effects of RGNps on hyperglycemia-induced oxidative stress in HUVEC cells, providing valuable insights into potential therapeutic applications. While comparing results from the MFC with those from animal studies might offer a more holistic perspective, the consistency between the MFC results and the cell culture plate emphasizes the reliability of the MFC data. One reason for the observed differences could be the dynamic flow system in the MFC, in contrast to the static condition of the plate. Several studies have validated the efficacy of microfluidic devices compared to traditional culture techniques^57^, further highlighting the potential advantages of using dynamic systems like the MFC.

## Conclusion

In this study, a single-channel MFC was fabricated by manipulating collagen levels to culture endothelial cells. Endothelial cells cultured in the MFC reacted to the dynamic flow and their morphology became spindle-like and elongated compared to the endothelial cells cultured in the static condition. As a result, the MFC, equipped with dynamic flow and an extracellular matrix protein surface modification, provides a more accurate and reliable platform for promoting natural cell morphology and functionality. HUVEC cells were cultured both on the MFC and on a cell culture plate, and then treated with RGNps, CGNps and free Resveratrol in certain concentrations. Cellular oxidative stress, associated with hyperglycemia was induced by high glucose levels treated in MFC and cell culture plate. Oxidative stress and endothelial dysfunction were confirmed by increasing ROS levels in HUVEC cells using the fluorescence detector DCFH-DA and observing MVEC cells. GNps were then fabricated as a low-toxicity carrier to transport Resveratrol as a potential antioxidant at 3 and 20 nm.

The biological application of Resveratrol has been expanded by synthesizing RGNps to overcome the limitations of free Resveratrol such as low water solubility, bioavailability and toxicity at high concentrations. In order to maintain effective antioxidant properties, minimal cytotoxicity was achieved. In the extracellular antioxidant analysis by DPPH, RGNps showed significant antioxidant properties compared to CGNps and free Resveratrol. Cell availability using Alamar-Blue indicated that RGNps treatment had the lowest toxicity at the highest concentrations compared to free Resveratrol. In addition, intracellular antioxidant analysis performed by DCFH-DA revealed that these nanoparticles were effective in reducing oxidative stress induced by high glucose concentration compared to CGNps and Resveratrol.

### Supplementary Information

Supplementary Figures.

## Data Availability

All data generated or analyzed during this study are included in this published article [and its supplementary information files].
