# Supplementary material for: Evaluating the antioxidant potential of resveratrol-gold nanoparticles in preventing oxidative stress in endothelium on a chip
Source: Sci Rep. 2023 Dec 1;13:21344. doi: 10.1038/s41598-023-47291-6 (PMC10696074; doi:10.1038/s41598-023-47291-6)
Supplement: Supplementary file 1 — Supplementary Figures. [file 41598_2023_47291_MOESM1_ESM.docx]

| 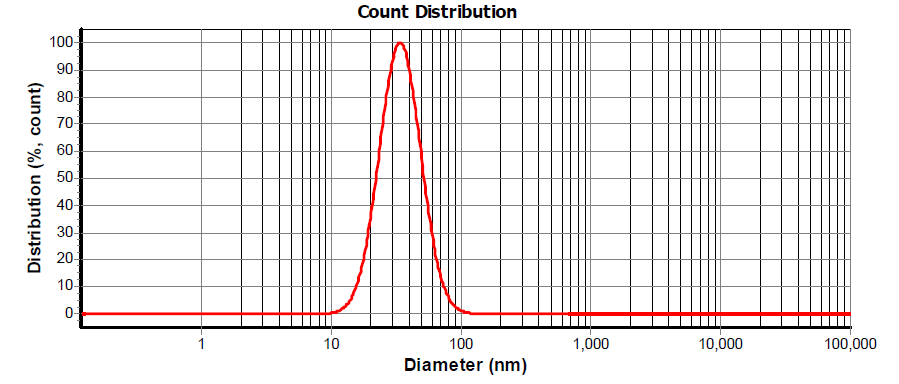  **(a)** |
| --- |
| 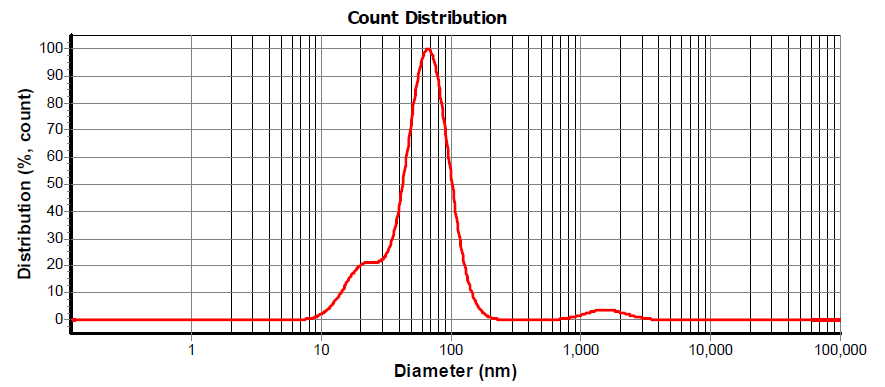  **(b)** |
| 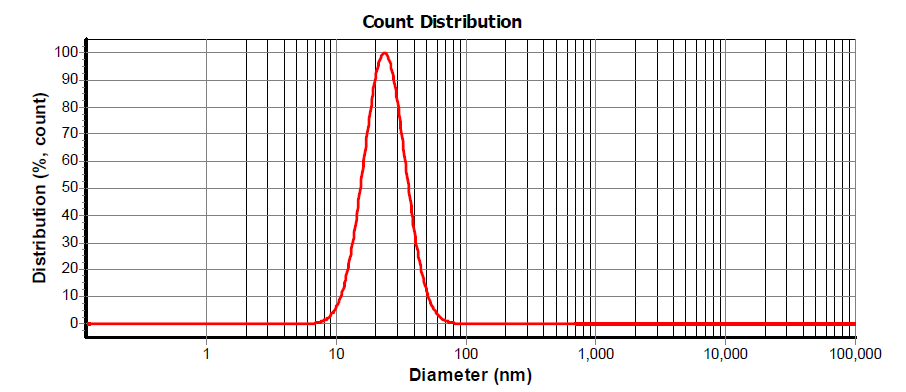  **(c)** |

Figure 1. DLS analysis of CGNps (a), RGNps (20 nm) (b), RGNps (3 nm) (c)

**(b)**

**(a)**

Figure 2. Zeta potential analysis of RGNps (20 nm) at -50.7±0.9 (a), RGNps (3 nm) at -46.4±3 (b), (mean±SD, n=3)

**Results of MFC cell culture**

The plasma treatment alone increased the hydrophilicity of the surface for cell attachment. Over time, when the cells are cultured in the channel, they were removed from the surface without collagen treatment easily. Therefore, surface modification with collagen can help cell attachment. As mentioned before, the suspension of HUVEC cells in DMEM was injected into the channel with FBS (10%). The appropriate cell density was about 2×10^6^ cells / ml. According to the image in figure 2, the cells were evenly distributed on the surface of the channel. In Figure 2 a, the cells completely attached to the surface after 6 h and after 24 h (Figure 2 b).

**(a)**

| 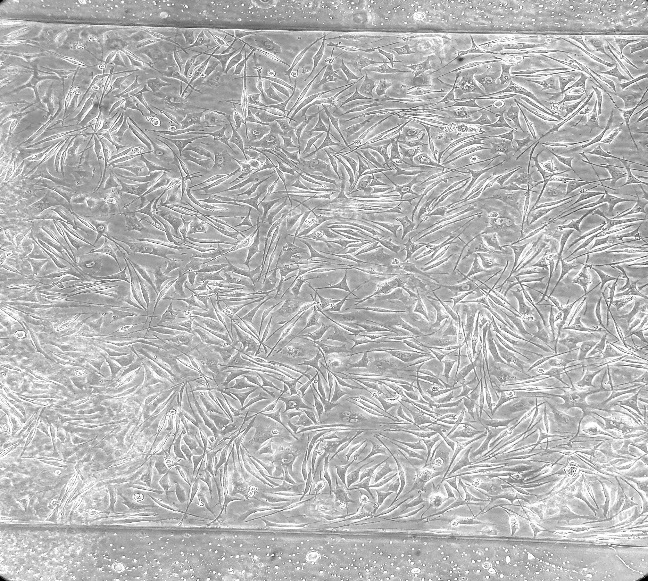  **(b)** | 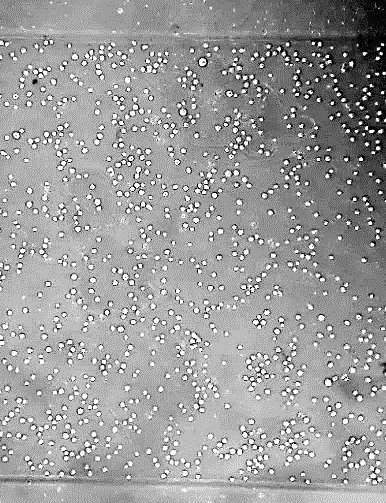 |
| --- | --- |

Figure 3. HUVEC cells in MFC channel after cell injection 10X after 6 h (a), and after 24h (b)

**(a)**

**(b)**

| 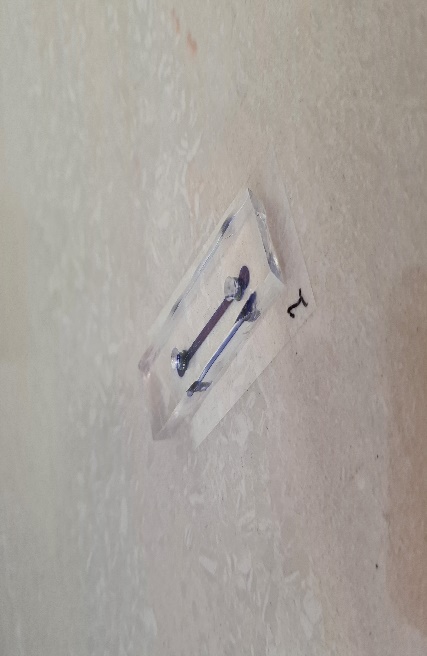 | 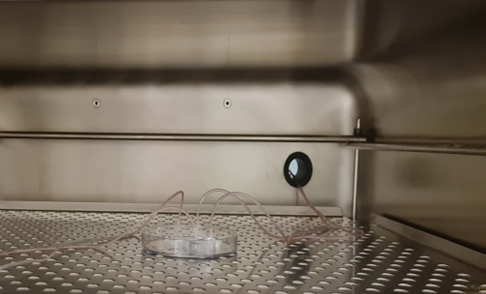 |
| --- | --- |

Figure 4. Fabricated MFC (a), MFC linked with cell culture medium (b)
